# A case of de novo splice site variant in *SLC35A2* showing developmental delays, spastic paraplegia, and delayed myelination

**DOI:** 10.1002/mgg3.814

**Published:** 2019-06-23

**Authors:** Sachiko Miyamoto, Mitsuko Nakashima, Tsukasa Ohashi, Takuya Hiraide, Kenji Kurosawa, Toshiyuki Yamamoto, Junichi Takanashi, Hitoshi Osaka, Ken Inoue, Takehiro Miyazaki, Yoshinao Wada, Nobuhiko Okamoto, Hirotomo Saitsu

**Affiliations:** ^1^ Department of Biochemistry Hamamatsu University School of Medicine Hamamatsu Japan; ^2^ Department of Pediatrics Niigata University Medical and Dental Hospital Niigata Japan; ^3^ Division of Medical Genetics Kanagawa Children's Medical Center Yokohama Japan; ^4^ Tokyo Women's Medical University Institute for Integrated Medical Sciences Tokyo Japan; ^5^ Department of Pediatrics and Pediatric Neurology Tokyo Women's Medical University, Yachiyo Medical Center Yachiyo Japan; ^6^ Department of Pediatrics Jichi Medical University Tochigi Japan; ^7^ Department of Mental Retardation & Birth Defect Research National Institute of Neuroscience National Center of Neurology & Psychiatry Japan; ^8^ Department of Molecular Medicine Osaka Women's and Children's Hospital Osaka Japan; ^9^ Department of Medical Genetics Osaka Women's and Children's Hospital Osaka Japan

**Keywords:** congenital disorders of glycosylation, delayed myelination, *SLC35A2*, spastic paraplegia, splice site variant

## Abstract

**Background:**

Congenital disorders of glycosylation (CDGs) are genetic diseases caused by pathogenic variants of genes involved in protein or lipid glycosylation. De novo variants in the *SLC35A2* gene, which encodes a UDP‐galactose transporter, are responsible for CDGs with an X‐linked dominant manner. Common symptoms related to *SLC35A2* variants include epilepsy, psychomotor developmental delay, hypotonia, abnormal facial and skeletal features, and various magnetic resonance imaging (MRI) findings.

**Methods:**

Whole‐exome sequencing was performed on the patient's DNA, and candidate variants were confirmed by Sanger sequencing. cDNA analysis was performed to assess the effect of the splice site variant using peripheral leukocytes. The X‐chromosome inactivation pattern was studied using the human androgen receptor assay.

**Results:**

We identified a de novo splice site variant in *SLC35A2* (NM_005660.2: c.274+1G>A) in a female patient who showed severe developmental delay, spastic paraplegia, mild cerebral atrophy, and delayed myelination on MRI, but no seizures. The variant led to an aberrant splicing resulting in an in‐frame 33‐bp insertion, which caused an 11‐amino acid insertion in the presumptive cytoplasmic loop. X‐inactivation pattern was random. Partial loss of galactose and sialic acid of the *N*‐linked glycans of serum transferrin was observed.

**Conclusion:**

This case would expand the phenotypic spectrum of *SLC35A2*‐related disorders to delayed myelination with spasticity and no seizures.

## 1 INTRODUCTION

The *Solute carrier family 35 member A2* gene (*SLC35A2*, OMIM#314375, HGNC ID: 11022; NM_005660.2), located at Xp11.23, encodes a UDP‐galactose transporter (UGT) belonging to the nucleotide‐sugar transporter family. The *SLC35A2* protein has 10 transmembrane domains and transports UDP‐galactose, which is a substrate for glycosylation, from cytoplasm into the Golgi or endoplasmic reticulum (ER) lumens (Hadley et al., 2014). To date, 53 de novo variants in *SLC35A2* have been identified in 62 patients with congenital disorders of glycosylation (CDGs) (MIM#300896) including 30 missense, four nonsense, 13 frameshift, one splice site, three in‐frame deletion, and two variants affecting translation initiation codon (Table S1) (Appenzeller et al., 2017; Bosch et al., 2016; Demos et al., 2017; Dörre et al., 2015; Hesse, Bevilacqua, Shankar, & Reddi, 2018; Hino‐Fukuyo et al., 2015; Kimizu et al., 2017; Kodera et al., 2013; Ng et al., 2013, 2019; Vals et al., 2019; Westenfield et al., 2018; Yates et al., 2018). These 62 patients comprised both men and women and most of the patients showed seizures leading to diagnosis of epileptic encephalopathy. The clinical spectrum of these patients includes developmental delay, microcephaly, dysmorphic features, ocular and skeletal abnormalities, infantile‐onset seizures, hypotonia, cerebral and cerebellar atrophy, and thin corpus callosum (Table S2) (Appenzeller et al., 2017; Bosch et al., 2016; Demos et al., 2017; Dörre et al., 2015; Hesse et al., 2018; Hino‐Fukuyo et al., 2015; Kimizu et al., 2017; Kodera et al., 2013; Ng et al., 2013, 2019; Vals et al., 2019; Westenfield et al., 2018; Yates et al., 2018).

Here, we present a patient with a splice site variant (c.274+1G>A) in *SLC35A2* showing severe developmental delay, spasticity, and delayed myelination of white matter. To the best of our knowledge, this is the first case of a splice site variant, in which aberrant mRNA splicing is demonstrated. We review the literature and discuss the expansion of the phenotypic spectrum of CDG related to *SLC35A2* variants.

## 2 SUBJECT AND METHODS

### 2.1 Ethical compliance

This study was approved by the Institutional Review Board Committee at Hamamatsu University School of Medicine and written informed consent was obtained.

### 2.2 Case report

**Figure 1:**
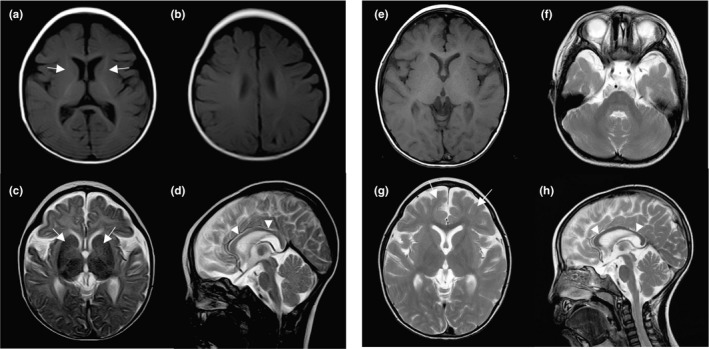
Brain magnetic resonance images of the patient at 7 months (a–d), and 3 years (e–h). T1‐weighted images (a, b and e), and T2‐weighted images (c, d, f, g and h). (a) The posterior limb of the internal capsule shows high intensity suggesting myelination, but the anterior limb of the internal capsule only partly shows high intensity (arrows). (b) The white matter of the centrum semiovale and the central sulcus circumference show slightly high intensity. (c) While the posterior limb of the internal capsule shows normal low intensity in T2‐weighted images, the anterior limb of the internal capsule shows high intensity, suggesting delayed myelination (arrows). (d) The corpus callosum is hypoplastic and shows isointensity, suggesting delayed myelination (arrowheads). (e) T1‐weighted image at 3 years shows high intensity in the subcortical and deep white matter, suggesting almost full myelination. (f) The white matter of the cerebellum still shows high intensity in T2‐weighted images. (g) Part of the subcortical white matter of the frontal lobes does not show low intensity (arrows), suggesting incomplete myelination in some parts. (h) The hypoplastic corpus callosum shows hypointensity, suggesting progress of myelination (arrowheads)

The 3‐year‐and‐3‐month‐old Japanese girl is the second child of nonconsanguineous healthy parents. She was born by cesarean section without neonatal asphyxia after an uneventful 38 weeks pregnancy. Her birth weight, length, and head circumference were 2,746 g (−0.6, standard deviation [*SD*]), 45 cm (−1.6 *SD*), and 33 cm (0 *SD*), respectively. There was no family history of neurodevelopmental disorders. Newborn screening was normal. G‐banded analysis showed a normal karyotype (46,XX). She often arched her back from 3 months of age, and developmental delay was noticed at 6 months of age because she had not acquired head control. Brain magnetic resonance imaging (MRI) at 7 months showed delayed myelination as myelination was only observed in the posterior limb of the internal capsule, optic radiation, corpus callosum, and middle cerebellar peduncle (Figure 1a–d). Brain MRI at 1 year and 8 months also showed delayed myelination in the subcortical and deep white matter (Figure S1a–d). Magnetic resonance spectroscopy focused on the basal ganglia and thalamus at 8 months was unremarkable. The auditory brain‐stem response (ABR) analysis delineated normal peak latency of wave I with 70 db stimulation. However, waves III and IV were not observed and prolonged latency and a very low amplitude of wave V was recognized.

Upon final examination at 3 years and 0 months, her height was 90 cm (−0.7 *SD*), and her weight 11.7 kg (−0.9 *SD*). She showed no obvious dysmorphic features of the face, but coxa valga. Her developmental milestones were severely delayed; she achieved head control at 10 months, rolling over at 1 year and 6 months, and sitting independently at 2 years. She spoke no meaningful words and was unable to crawl and stand (developmental quotient = 22, the Enjoji Scales of Infant Analytical Development). She showed spasticity with lower‐limb dominance but no rigidity, involuntary movements, muscle atrophy, and weakness. Deep tendon reflexes of the lower limbs were brisk and the Babinski reflex was bilaterally positive. Blood tests, coagulation tests, and urine were all normal. Brain MRI at 3 years revealed progress of myelination except for a part of the subcortical white matter of the frontal lobes, and a part of deep white matter of the cerebellum. Thinning of the corpus callosum and mild cerebral atrophy were also observed (Figure 1e–h). The ABR showed bilateral prolonged peak latency of wave V and interpeak latency of waves I–V. The visual evoked potential showed clear latency of P100 at 95 ms. Short latency somatosensory evoked potentials showed abnormal, central somatosensory conduction time on the stimulation of the left (8.0 ms) and right (12.6 ms) median nerves, indicating clear prolongation on the right. Electroencephalography did not reveal obvious abnormalities.

### 2.3 Whole‐exome sequencing

Genomic DNA of the proband was extracted from peripheral blood leukocytes and captured using a SureSelect Human All ExonV6 kit (Agilent Technologies, Santa Clara, CA). Sequencing was performed by NextSeq500 (Illumina, San Diego, CA) with 150 bp paired‐end reads. Exome data processing, variant calling, and variant annotation were performed as previously described (Hiraide et al., 2018). Candidate variants detected by WES were confirmed by Sanger sequencing. Biological parentage was confirmed by analyzing 10 microsatellite markers.

### 2.4 Reverse transcription polymerase chain reaction

**Figure 2:**
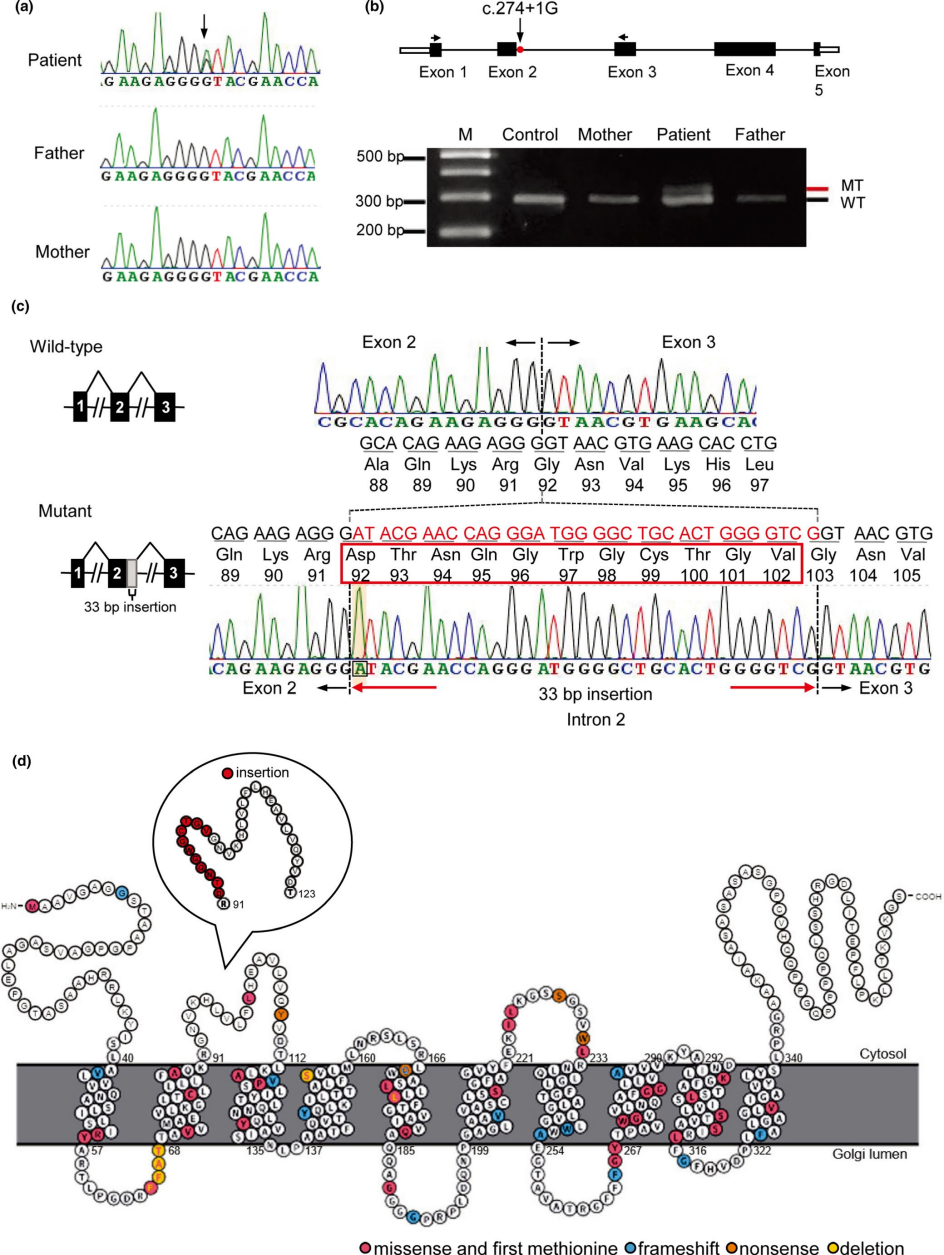
A de novo splice site variant in *SLC35A2* (OMIM#314375, HGNC ID: 11022; NM_005660.2) and its effects on splicing. (a) Sanger sequencing using family trio samples confirmed that the c.274+1G>A variant occurred de novo (arrow). (b) Schematic representation of the gene structure of *SLC35A2* (top). White and black boxes denote the 5’ or 3’ untranslated region and coding exons, respectively. RT‐PCR was performed using target‐specific primers designed at exons 1 and 3 (arrows). Two different‐sized PCR products were observed only in the patient (bottom). The lower band is the wild‐type (WT) transcript, and the upper band is the mutant (MT). (c) Sequence of WT and mutant amplicons clearly shows that 33 nucleotides (the 5′ end of intron2, red characters) are inserted at the exon boundary between exons 2 and 3 (dashed lines) leading to in‐frame 11 amino acid insertion (red box). (d) Topology prediction of the human UDP‐galactose transporter (Hadley et al., 2014; Kelley, Mezulis, Yates, Wass, & Sternberg, 2015; Omasits, Ahrens, Muller, & Wollscheid, 2014). Location of the previously reported missense (include first methionine), frameshift, nonsense, and in‐frame deletion variants are shown in magenta, blue, orange, and yellow, respectively. The inset indicates location of in‐frame 11 amino acid insertion at 92nd amino acid residue (red)

Total RNA from family trio and one healthy control was extracted from whole blood using a Monarch Total RNA Miniprep kit (NEW ENGLAND BioLabs, Ipswich, MA) and subjected to reverse transcription using the PrimeScript RT reagent kit (TAKARA BIO, Kusatsu, Shiga) according to the manufacturer's protocol. We designed target‐specific primers for *SLC35A2*(NM_005660.2) (Figure 2c, Table S3) and 2 μl of cDNA was used for polymerase chain reaction (PCR). PCR products were separated by electrophoresis on a 3% agarose gel and extracted from gels and cloned into pGEM‐T easy vectors (Promega, Madison, WI) using Ligation high (TOYOBO, Osaka, Japan), then transfected into DH5α competent cells (TOYOBO). Competent cells were spread on LB plates containing ampicillin and incubated at 37°C overnight. We performed colony PCRs on randomly selected well‐isolated colonies, and products of the colony PCR were sequenced.

### 2.5 Plasmid construction

A full‐length wild‐type (WT) human *SLC35A2* cDNA (393 amino acids, GenBank accession number NM_001042498.2) (Kodera et al., 2013) was cloned into the p3xFlag‐CMV‐7.1 vector (Sigma‐Aldrich, St. Louis, MO) to produce amino‐terminally Flag tagged SLC35A2. Site‐directed mutagenesis was performed using a KOD‐Plus‐Mutagenesis kit (Toyobo, Osaka, Japan) according to the manufacturer's protocol. The mutant cDNA was confirmed by Sanger sequencing.

### 2.6 Cell culture, transfection, and immunoblotting

HEK293T human embryonic kidney cells were grown in Dulbecco's minimum essential medium (Wako, Tokyo, Japan) supplemented with 10% fetal bovine serum, 100 units/ml penicillin, and 100 μg/ml streptomycin (all from Wako) at 37°C in a 5% CO2 incubator. Cells in 6‐well plate were transfected with 3 μg WT and mutant *SLC35A2* expression vectors using polyethylenimine hydrochloride reagent (PEI MAX‐40,000, Polysciences, Inc, Warrington, PA 24765‐1). At 24‐hr posttransfection, cells were washed with ice‐cold PBS and lysed with RIPA lysis buffer (150 mM NaCl, 50 mM Tris HCl [pH 7.4], 1 mM EDTA. 1% NP40, 0.5% sodium deoxycholate, 0.1% SDS, 1xPhoshatase inhibitor, 1 mM PMSF) and then homogenized by 21G needle. Samples were separated by SDS‐PAGE and analyzed by immunoblotting using anti‐FLAG (1:10,000 dilution; clone M2; Sigma‐Aldrich) antibody. The secondary antibody against anti‐FLAG and anti‐βactin antibodies were goat anti‐mouse antibody conjugated with horseradish peroxidase (1:5,000 dilution). The membrane was treated with Clarity Western blot ECL substrate (BioRad, Hercules, CA) and signals were captured using a Fusion FX imaging system (Fusion FX, Vilber, France).

### 2.7 X‐chromosome inactivation analysis

The X‐chromosome inactivation (XCI) pattern was studied using the human androgen receptor (HUMARA) assay (Allen, Zoghbi, Moseley, Rosenblatt, & Belmont, 1992). Family trio DNA samples were incubated overnight at 37°C with *Hpa* II and *Hha* I. The same samples were subjected to mock digestion without the restriction enzymes. The digested and mock‐digested samples were amplified by PCR using a fluorescently labeled primer set for targeting the short tandem repeat of the HUMARA locus (Table S3). The PCR products were analyzed on a 3130 Genetic Analyzer (Applied Biosystems, Foster City, CA). Fragment analysis was performed by Peak Scanner 2 software (Applied Biosystems). The experiments were repeated three times.

## 3 RESULTS

**Figure 3:**
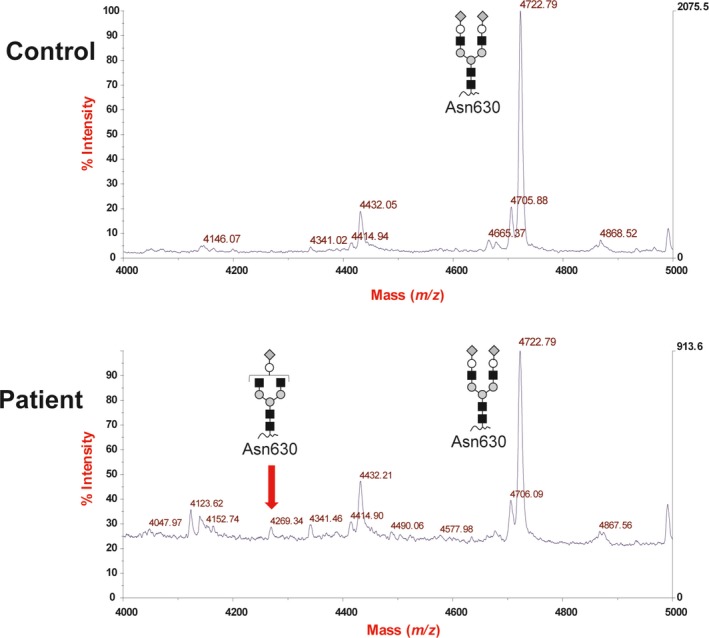
MALDI‐MS spectra of *N*‐linked glycans from serum transferrin of a control and the patient at 3 years. Partial loss of both galactose and sialic acid of the *N*‐linked glycans was observed in the patient. The symbols representing sugar residues are as follows: gray diamonds, sialic acid; closed circles, galactose; black squares, *N*‐acetylglucosamine; gray circles, mannose; and gray triangles, fucose

We searched for possible pathogenic variants using WES data and identified a canonical splice site variant in *SLC35A2* (NM_005660.2: c.274+1G>A). This variant was not observed in the gnomAD (http://gnomad.broadinstitute.org/), 2KJPN (https://ijgvd.megabank.tohoku.ac.jp/), HGVD databases (http://www.hgvd.genome.med.kyoto-u.ac.jp/), or our in‐house 218 control exome data. Sanger sequencing confirmed that this variant occurred de novo (Figure 2a). The variant is considered pathogenic according to ACMG Standards and Guidelines (PVS1, PS1, PM2, and PP3) (Richards et al., 2015). To examine the functional impact of this variant on mRNA splicing, we performed reverse transcription polymerase chain reaction (RT‐PCR) on total RNA extracted from peripheral leukocytes. Two different‐sized products were amplified only in the patient (Figure 2b), and sequencing of cloned PCR products showed that the upper mutant band had retention of a 33‐bp 5′ sequence of intron 2, which causes an in‐frame 11 amino acid insertion in the presumptive cytoplasmic loop of the SLC35A2 protein (Figure 2c,d). To confirm whether this mutant transcript produces a protein in human cells, HEK293T cells were transfected with WT and mutant *SLC35A2* expression vectors. Immunoblot analysis showed that a slightly higher molecular weight product than WT was observed in cell lysates transfected with mutant *SLC35A2* vectors (Figure S2). The XCI analysis showed a random XCI pattern in the patient's leukocytes (79:21, Figure S3). Serum transferrin at 3 years of age showed partial loss of galactose and sialic acid of the *N*‐linked glycans in the patient (Figure 3).

## 4 DISCUSSION

*SLC35A2*‐related disorders show a wide phenotypic spectrum and comparing clinical findings of our patient with those of previously reported patients allows us to identify both common and different features (Table S2) (Appenzeller et al., 2017; Bosch et al., 2016; Demos et al., 2017; Dörre et al., 2015; Hesse et al., 2018; Hino‐Fukuyo et al., 2015; Kimizu et al., 2017; Kodera et al., 2013; Ng et al., 2013, 2019; Vals et al., 2019; Westenfield et al., 2018; Yates et al., 2018). Most cases had neurological disorders including developmental delay (100%, 61/61), seizures (84%, 52/62), and hypotonia (92%, 54/59). Various dysmorphic features such as facial dysmorphism (86%, 50/58), ocular abnormalities (75%, 42/56), and skeletal abnormalities (83%, 43/52), were observed. In brain imaging findings, cerebral atrophy (50%, 13/26), cerebellar atrophy (46%, 26/56), thin corpus callosum (39%, 23/56), and delayed/hypo myelination (58%, 15/26) were relatively common. The present case showed many of the common features of *SLC35A2*‐related disorders but did not show seizures, facial dysmorphism, or hypotonia. Instead, she showed spastic paraplegia. Considering the delayed myelination and absence of seizures observed in this patient, it is possible that neuronal dysfunction may be mild compared with that of previous cases leading to lack of hypotonia, and spasticity caused by cerebral delayed myelination become evident. Delayed myelination was also suggested by prolonged latency of wave V and central somatosensory condition time. Thus, this case would expand the phenotypic spectrum of *SLC35A2*‐related disorders to delayed myelination with spasticity.

Glycosylated proteins such as myelin basic protein and myelin‐associated glycoprotein, and glycosylated lipids such as galactosylceramide (GalCer) play an important role in maintaining nerve function (Han, Myllykoski, Ruskamo, Wang, & Kursula, 2013; Jackman, Ishii, & Bansal, 2009). These glycoconjugates are synthesized in the lumen of the ER and Golgi apparatus. This glycosylation procedure requires nucleotide sugars activated by the addition of mono‐ or diphosphonucleotides (UDP, GDP, or CMP), which are synthesized in the cytosol or the nucleus. Nucleotide sugar transporters transport nucleotide sugars into the ER or Golgi apparatus (Ishida & Kawakita, 2004). Functional impairment of UGT encoded by *SLC35A2* may cause disability of UDP‐galactose transportation leading to deficiency of glycoproteins and GalCer (Freeze, 2013). In the brain of mammals, GalCer is a major component of the myelin sheath, a multilamellar structure consisting of a spirally wrapped extension of the plasma membrane of oligodendrocytes (Ichikawa & Hirabayashi, 1998). Therefore, decrease in the synthesis of GalCer and glycoproteins would cause delayed myelination, which is one of the major clinical findings of this case.

Carbohydrate‐deficient transferrin (CDT) analysis is useful for the initial screening of CDGs because the defect of sialic acid and galactose of glycans will occur in cases of SLC35A2‐CDGs (Lacey, Bergen, Magera, Naylor, & O'Brien, 2001; Ng et al., 2013). In this case, we analyzed *N*‐glycans of serum transferrin after genetic diagnosis. The result of mass spectrometry analysis showed partial loss of both galactose and sialic acid of the *N*‐linked glycans in the patient, which was consistent with the genetic diagnosis of SLC35A2‐CDGs. Although CDT screening is an important technique for clinical CDGs diagnosis, the positive rate of CDT abnormalities in cases of SLC35A2‐CDGs was not so high (17/51, 33%). Therefore, it is considered that combination of genetic screening and molecular diagnosis would contribute to the improvement of diagnostic yield of SLC35A2‐CDGs.

Interestingly, it has been recently reported that somatic variants in *SLC35A2* in human brain tissues are associated with nonlesional focal epilepsy or mild malformation of cortical development (Sim et al., 2018; Winawer et al., 2018). Therefore, lesion specific de novo somatic variants in *SLC35A2* could be one of the etiologies of neurological disorders in addition to germline de novo variants. These facts also highlight essential roles of UGT in normal brain function.

In summary, we report a de novo splice site variant in *SLC35A2* in a patient showing delayed myelination with spasticity but no seizures. A pathogenic *SLC35A2* variant was never expected based on clinical features, suggesting that whole‐exome sequencing has great utility for differential diagnosis of congenital neurodevelopmental disorders.

## CONFLICTS OF INTEREST

The authors declare no conflict of interest.
