# Supplementary material for: A case of de novo splice site variant in SLC35A2 showing developmental delays, spastic paraplegia, and delayed myelination
Source: Mol Genet Genomic Med. 2019 Jun 23;7(8):e814. doi: 10.1002/mgg3.814 (PMC6687661; doi:10.1002/mgg3.814)
Supplement: Supplementary file 1 [file MGG3-7-e814-s001.docx]

**Supporting information**

**
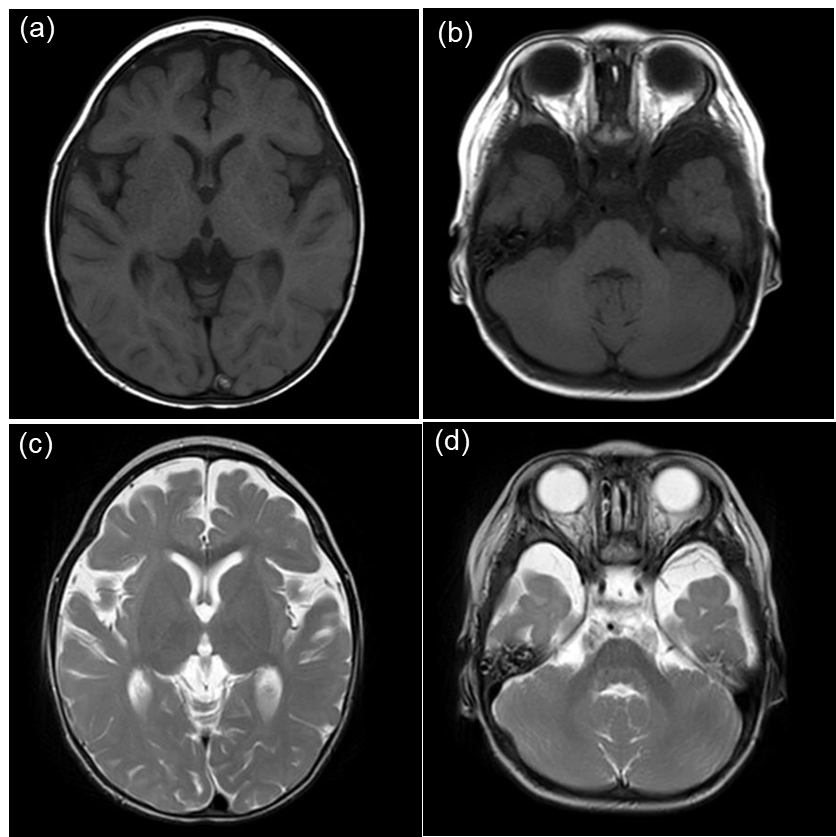
**

**Supplementary Figure S1** Brain magnetic resonance images of the patient at the age of 1 year and 8 months. T1-weighted images (a, b), and T2-weighted images (c, d). (a) The subcortical frontal lobe shows slight myelination delay. (b) The cerebellum white matter shows high intensity, suggesting progress of myelination (arrows). (c) In the T2-weighted image, the corpus callosum shows low intensity, suggesting progress of myelination (arrows). However, the subcortical and deep white matter show iso- or high intensity, suggesting delayed myelination. (d) The cerebellum white matter still shows high intensity (arrows).

Neg

WT

Mut


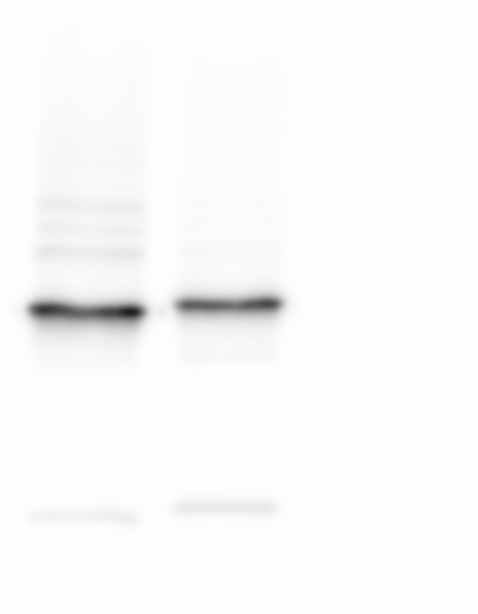


45kDa

35kDa

**Supplementary Figure S2** Western blot analysis of SLC35A2 protein. Representative western blots using extracts from HEK293T cells transiently expressing SLC35A2 is shown. WT, wild-type; Mut, Mutant; Neg, negative control with untransfection.


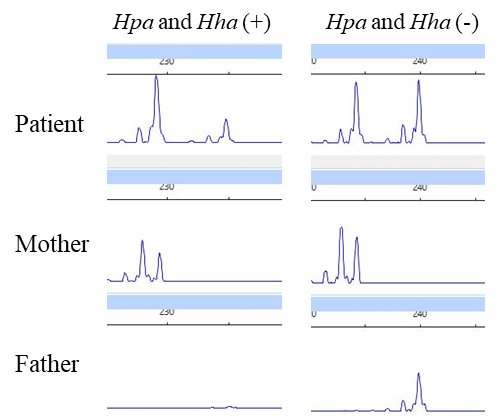


|  | 1st | 2nd | 3rd | Average |
| --- | --- | --- | --- | --- |
| Patient | Random | Random | Skewing | Random |
|  | (77.1:22.9) | (76.8:23.2) | (85.3:14.7) | (79:21) |
| Mother | Random | Random | Random | Random |
|  | (52.3:47.7) | (54.8:45.2) | (56:44) | (54:46) |

**Supplementary Figure S3** Summary of X-chromosome inactivation study.

**Supplementary Table S1** Summary of pathogenic *SLC35A2* variants in 63 patients with CDG.

| Individuals | | Inheritance | Variant | |
| --- | --- | --- | --- | --- |
| No | Authors |  | Variant | Amino acid |
| 1 | This case | *de novo* | c.274+1G>A |  |
| 2 | Ng et al.(2013) | *de novo* (mosaic) | c.15_91+48delinsA | p.Gly8Serfs*9 |
| 3 |  | *de novo* | c.3G>A | p.Met1? |
| 4 |  | *de novo* (mosaic) | c.991G>A | P.Val331Ile |
| 5 | Kodera et al. | *de novo* | c.433_434del | p.Tyr145Profs*76 |
| 6 |  | *de novo* | c.972delT | p.Phe324Leufs*25 |
| 7 |  | *de novo* | c.638C>T | p.Ser213Phe |
| 8 | Dörre et al. | *de novo* | c.797G>T | p.Gly266Val |
| 9 | Bosch et al. | *de novo* | c.800A>G | p.Tyr267Cys |
| 10 | Kimizu et al. | *de novo* | c.950delG | p.Gly317Alafs*32 |
| 11 | EuroEPINOMICS et al. | *de novo* | c.683C>A | p.Ser228* |
| 12 |  | *de novo* | c.502C>T | p.Gln168* |
| 13 | Yates TM et al. | *de novo* | c.889A>G | p.Lys297Glu |
| 14 |  | *de novo* | c.327T>G | p.Tyr109* |
| 15 |  | *de novo* | c.195C>A | p.Phe65Leu |
| 16 |  | *de novo* | c.515T>C | p.Leu172Pro |
| 17 |  | *de novo* | c.923C>T | p.Ser308Phe |
| 18 | Hesse et al. | N/A | c.695G>A | p.Trp232* |
| 19 | Hino-Fukuyo et al. | *de novo* | c.884G>C | p.Gly282Arg |
| 20 | Westenfield et al. | *de novo* (mosaic) | c.991G>A | p.Val331Ile |
| 21 | Vals et al. | *de novo* | c.670C>T | p.Leu224Phe |
| 22 |  | *de novo* | c.262G>C | p.Ala88Pro |
| 23 |  | *de novo* | c.753delG | p.Trp251Cysfs*98 |
| 24 |  | *de novo* | c.124del | p.Val42Cysfs*53 |
| 25 |  | *de novo* | c.923C>T | p.Ser308Phe |
| 26 |  | *de novo* (mosaic) | c.818G>A | p.Gly273Asp |
| 27 |  | *de novo* | c.389A>G | p.Tyr130Cys |
| 28 |  | *de novo* | c.991G>A | p.Val331Ile |
| 29 |  | *de novo* | c.698T>C | p.Leu233Pro |
| 30 |  | *de novo* | c.841G>A | p.Gly281Ser |
| 31 |  | *de novo* | c.841G>C | p.Gly281Arg |
| 32 |  | *de novo* (mosaic) | c.164G>C | p.Arg55Pro |
| 33 | Demos et al. | *de novo* | c.466_468delTCC | p.Ser156del |
| 34 | Ng et al.(2019) | *de novo* | c.523_525del | p.Leu175del |
| 35 |  | *de novo* | c.193_204del | p.Phe65_Thr68del |
| 36 |  | *de novo* | c.816G>A | p.Trp272Ter |
| 37 |  | *de novo* | c.348del | p.Val117Cysfs*27 |
| 38 |  | *de novo* | c.562G>A | p.Gly188Ser |
| 39 |  | *de novo* | c.497_501dup | p.Gln168Glyfs*183 |
| 40 |  | *de novo* | c.168C>A | p.Tyr56Ter |
| 41 |  | *de novo* | c.274+2T>C | p.? |
| 42 |  | *de novo* | c.617del | p.Val206Alafs*143 |
| 43 |  | *de novo* | c.856del | p.Ala286Leufs*63 |
| 44 |  | *de novo* | c.547C>T | p.Gln183Ter |
| 45 |  | *de novo* | c.908T>C | p.Leu303Pro |
| 46 |  | *de novo* | c.795del | p.Phe265Leufs*84 |
| 47 |  | *de novo* | c.935C>A | p.Ser312Tyr |
| 48 |  | *de novo* | c.991G>A | P.Val331Ile |
| 49 |  | *de novo* | c.211G>A | p.Val71Met |
| 50 |  | *de novo* | c.346G>C | p.Ala116Pro |
| 51 |  | *de novo* | c.302T>C | p.Leu101Pro |
| 52 |  | *de novo* | c.818G>A | p.Gly273Asp |
| 53 |  | *de novo* | c.353C>G | p.Pro118Arg |
| 54 |  | *de novo* | c.747_757dup | p.Ala253Glyfs*100 |
| 55 |  | *de novo* | c.523C>T | p.Leu175Phe |
| 56 |  | *de novo* | c.164G>C | p.Arg55Pro |
| 57 |  | *de novo* | c.698T>C | p.Leu233Pro |
| 58 |  | *de novo* | c.1A>G | p.Met1? |
| 59 |  | *de novo* | c.569dup | p.Gly191Argfs*31 |
| 60 |  | *de novo* | c.944T>C | p.Leu315Pro |
| 61 |  | *de novo* | c.502C>T | p.Gln168Ter |
| 62 |  | *de novo* | c.389A>G | p.Tyr130Cys |
| 63 |  | *de novo* | c.245G>T | p.Cys82Phe |

*SLC35A2*(OMIM#314375, HGNC ID: 11022; NM_005660.2)

**Supplementary Table S2** Clinical summary of patients with pathogenic *SLC35A2* variants.

|  | This case | Previous reported cases |
| --- | --- | --- |
| **General information** |  |  |
| Gender | F | F:M = 56:6 |
| **Neurological phenotype** |  |  |
| Seizures | - | 84% (52/62) |
| Developmental delay | + | 100% (61/61) |
| Hypotonia | - (Spasticity) | 92% (54/59) |
| **Extraneurological phenotype** |  |  |
| Skeltal findings | + | 83% (43/52) |
| Ocular findings | + (Esotropia) | 75% (42/56) |
| Facial dysmorphisms | - | 86% (50/58) |
| **Brain MRI findings** |  |  |
| Cerebral atrophy | + | 50% (13/26) |
| Cerebellar atrophy | - | 46% (26/56) |
| Thin corpus callosum | + | 39% (22/56) |
| Delayed/hypo myelination | + | 58% (15/26) |
|  |  |  |

F, Female; M, Male

**Supplementary Table S3** PCR conditions and primer sequences.

*SLC35A2* (OMIM#314375, HGNC ID:11022; NM_005660.2) variant confirmation

| Exons | Product Size (bp) | Forward primer (5'>3') | Reverse primer (5'>3') |
| --- | --- | --- | --- |
| Ex2 | 378 | AGGGTCCTGGGTGAGAAAGA | AGACACTCCTGAAGCAAACCA |

The PCR reaction is comprised of 30 cycles in the following conditions: 5 cycles in 98°C for 10 sec, 62°C for 30sec, 68°C for 30sec,

5 cycles in 98°C or 10 sec, 60°C for 30sec, 68°C for 30sec, 5 cycles in 98°C for 10 sec, 58°C for 30sec, 68°C for 30sec, 15 cycles in 98°C for 10 sec, 56°C for 30sec, 68°C for 30sec.

RT-PCR analysis

| Amplicons | Product Size (bp) | Forward primer (5'>3') | Reverse primer (5'>3') |
| --- | --- | --- | --- |
| Ex1-3 | 301 | GGTTGGGGCTGGTGGTTC | CTCATGGAGGAAGAGAACCAGG |

The PCR reaction is comprised of 30 cycles in the following conditions: 98°C for 10 sec, 68°C for 30sec.

X inactivation analysis

| Assays | Product Size (bp) | Forward primer (5'>3') | Reverse primer (5'>3') |
| --- | --- | --- | --- |
| HUMARA | 240 | TCCAGAATCTGTTCCAGAGCGTGC | CTCTACGATGGGCTTGGGGAGAAC |

The PCR reaction is comprised of 30 cycles in the following conditions: 98°C for 10 sec, 68°C for 30sec.
